# Do cleaning organisms reduce the stress response of client reef fish?

**DOI:** 10.1186/1742-9994-4-21

**Published:** 2007-10-18

**Authors:** Redouan Bshary, Rui F Oliveira, Tânia SF Oliveira, Adelino VM Canário

**Affiliations:** 1 Department of Biology, University of Neuchâtel, Emile-Argand 11, 2009 Neuchâtel, Switzerland; 2 Insituto Superior de Psicologia Aplicada, R. Jardim do Tabaco 34, 1149-041 Lisboa, Portugal; 3 Centro de Ciências do Mar, Universidade do Algarve, Campus de Gambelas, 8000-117 Faro, Portugal

## Abstract

**Background:**

Marine cleaning interactions in which cleaner fish or shrimps remove parasites from visiting 'client' reef fish are a textbook example of mutualism. However, there is yet no conclusive evidence that cleaning organisms significantly improve the health of their clients. We tested the stress response of wild caught individuals of two client species, *Chromis dimidiata *and *Pseudanthias squamipinnis*, that had either access to a cleaner wrasse *Labroides dimidiatus*, or to cleaner shrimps *Stenopus hispidus *and *Periclimenes longicarpus*, or no access to cleaning organisms.

**Results:**

For both client species, we found an association between the presence of cleaner organisms and a reduction in the short term stress response of client fish to capture, transport and one hour confinement in small aquaria, as measured with cortisol levels.

**Conclusion:**

It is conceivable that individuals who are more easily stressed than others pay a fitness cost in the long run. Thus, our data suggest that marine cleaning mutualisms are indeed mutualistic. More generally, measures of stress responses or basal levels may provide a useful tool to assess the impact of interspecific interactions on the partner species.

## Background

The impact of cleaning organisms on the health of so called clients, from which they remove parasites and other material, has been subject of considerable research particularly in the marine environment (Reviews by [1-4]). An early experiment [5] in which all organisms known to clean from reefs were removed suggested that cleaning mutualism is of major importance for fish health and for the structuring of fish communities in coral reefs. Within days after the removal, many client species had migrated from the reefs, and remaining fish were often infected with fungus [5]. Unfortunately, no control reefs were sampled. The issue became controversial when several attempts to repeat the original study failed to produce similar results [6-9]. These failures promoted the proposal of an alternative hypothesis, namely that cleaner fish exploit the sensory system of their clients by being in almost constant body contact with their pelvic fins [2,10]. In this scenario, clients seek cleaners to receive a tactile reward. Losey [2,10] assumed that this tactile reward has no influence on client fitness and that interactions are overall more or less neutral to client fitness.

Recent cleaner removal and for the first time cleaner introduction experiments, conducted over large time scales and controls, have finally been able to show that cleaner fish presence promotes local reef fish diversity [11,12]. Studies looking directly at parasite distribution on clients have found evidence for a reduction of parasite load and cleaner preference for more parasitized clients, which also supports the original view that interactions are mutualistic [13-16]. In particular, clients had a fourfold increase in parasite loads within 12 h when deprived of access to cleaner wrasses, *Labroides dimidiatus*, in a field experiment [17]. A further laboratory experiment revealed that the same cleaner species was actually searching for parasites during interactions rather than scraping the clients' surface opportunistically [18]. Stomach analyses using new techniques also showed that cleaners eat about a 1000 parasites per day [19]. In addition to the benefits of parasite removal, the tactile stimulation that cleaners provide with their pectoral and pelvic fins [20,21] might bear additional positive effects, i. e. through calming, similar to massage in humans [22]. However, cleaning interactions do not only yield benefits to clients. Several costs of cleaning have been identified for clients, arising from the consumption of healthy client tissue [23], client time loss, and at least for some client species potential costs from having to enter foreign territories, or leaving the one's own territory empty for invasion by conspecifics [4]. Combining the fitness consequences of each positive and each negative effect into a single net outcome still has to be achieved.

Here we use a new method that may give insights about the net results of cleaning mutualism in particular and interspecific interactions in general. We assess the impact of the cleaning wrasse *L. dimidiatus *on cortisol levels as a physiological indicator of the stress response of two client species, black & white chromis, *Chromis dimidiata *and threatfin anthias, *Pseudanthias squamipinnis*, at Ras Mohammed National Park, Egypt. In this area, a patchy distribution of reef and cleaner fish ensures that in both client species, some individuals have access to a cleaner fish while others do not. We could thus collect client with and without access to cleaners directly from the field.

Cortisol is the main glucocorticoid produced by the teleost interrenal tissue in response to a stressor, and thus its circulating levels have been commonly used as an indicator of stress exposure in fish studies [24,25]. It should be noted here that the short-term response to a stressor is not inherently detrimental; on the contrary, it should be seen as adaptive in the sense that it prepares the organism to successfully cope with that challenge, by readjusting the metabolism of the organism accordingly (e.g. energy mobilisation). However, repeated or prolonged activation of the stress response (i.e. chronic stress) becomes maladaptive due to the potential pathophysiological effects of a sustained stress response (e.g. immunosupression, inhibition of reproduction, etc.) [26]. Two concepts, allostasis and allostatic load [27,28], have been introduced in the stress literature to conceptualize this paradoxical trade-off between short-term benefits and long-term negative consequences of the stress response. Allostasis, meaning "achieving stability through change" [27], is a key process in maintaining homeostasis in adverse environmental conditions since it allows the organism to reset internal critical variables to changes in environmental demands [29]. The accumulated costs to the body of repeated activation of the allostatic response is named allostatic load and results from three types of physiological responses: the frequency and magnitude of the response, the chronic activation and failure to shut-off these responses and the failure to respond to the challenge [30]. Therefore, measuring the magnitude of a response to a stressor is potentially more informative than measuring baseline stress levels, to make inferences about the allostatic load of an individual. If one considers an equal exposure to stressors stress responders are expected to experience a higher increase in their allostatic load and consequently decreased fitness. This assumption has recently been confirmed empirically in the European white stork (*Ciconia ciconia*), where individuals with higher stress-induced corticosterone levels have lower probability of survival and of recruitment into the breeding population than low stress responders [31].

In this study we have used a confinement stress paradigm to measure the cortisol response to a stress protocol. Fish were captured with a hand-net, brought to shore and placed inside a small aquarium for 1 h, after which the fish were released back into the sea and the cortisol levels were measured from the holding-water. The rationale of our study is based on the assumption that individuals that are more responsive to an acute stress situation will increase their allostatic load more rapidly than those that are less responsive. As mentioned above, this assumption is supported by psychoneuroimmunology literature that links not only the frequency but also the magnitude of an individual's stress responses to its health [30]. We therefore predict that if clients obtain a net health benefit from cleaning interactions, individuals with access to cleaners should have a lower stress response than those individuals that had no access to cleaners. We also sampled chromis and anthias from few places where cleaner shrimps, *Periclimenes longicarpus *and *Stenopus hispidus *were present. Little is known about their importance for the health of reef fish but it has been shown recently that two cleaner shrimp species in Australia eat ectoparasites [32]. Our data give some further indication about the importance of cleaner shrimps for the health of coral reef fishes.

## Results

### Stress response of *C. dimidiata*

**Figure 1:**
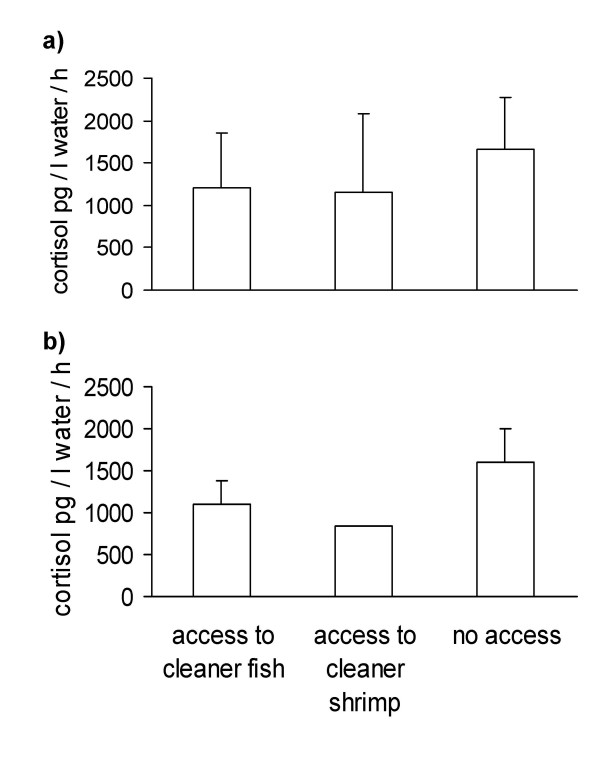
Variation (mean and STD) of cortisol responses to restraining stress in chromis individuals with and without access to cleaner organisms (cleaner wrasse or cleaner shrimps): (a) individual values; (b) median values per reef patch (no STD for second column as n = 2).

*C. dimidiata *individuals differed significantly between test groups in their stress response (Kruskal-Wallis-Test, H_(2, *n *= 46) _= 8.2, p < 0.05). Individuals from reef patches without cleaning organisms excreted more cortisol than individuals that had access to either cleaner fish or cleaner shrimps (post-hoc multiple comparisons, both p < 0.05, Fig. 1a). Individuals from the same reef patch might not be independent of each other in their stress response since they share the same social environment. Therefore we also used the median value of individuals for each reef patch. Again, individuals with no access to cleaning organisms secreted significantly more cortisol (Kruskal-Wallis-Test, H_(2, *N *= 16) _= 7.6 p < 0.05; post-hoc multiple comparisons: both p < 0.05, Fig. 1b).

### Stress response of *P. squamipinnis*

**Figure 2:**
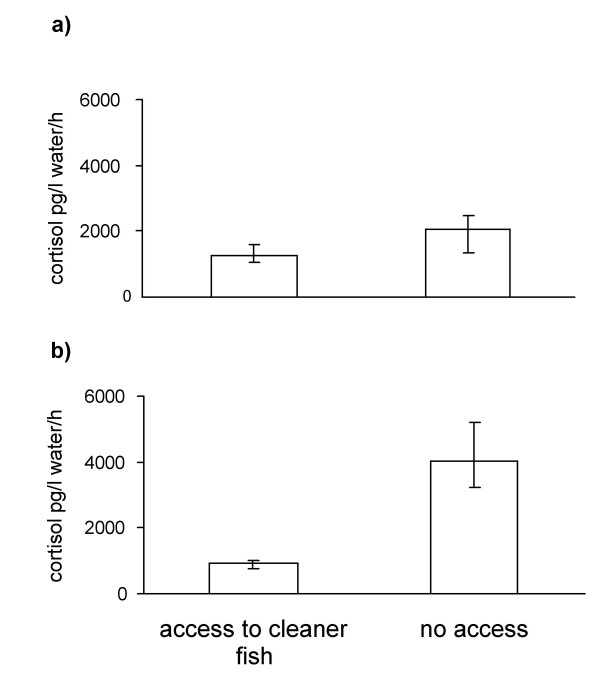
Variation (median and upper and lower quartile values) of cortisol responses to restraining stress in anthias individuals with and without access to cleaner wrasses. 2a: matched pair design, 14 individuals measured twice, once with access to a cleaner wrasse, once without access to a cleaner wrasse. 2b) independent samples of individuals living at the same reef patch, first group caught while a cleaner wrasse was present, second group caught 14 days after cleaner fish removal.

Paired data: 6 out of 9 *P. squammipinnis *males that originally lived on a reef patch without a cleaner fish had a lower stress response after the addition of a cleaner fish. In contrast, 3 out of 4 males that originally had access to a cleaner fish had a higher stress response after the removal of the cleaner fish. Taken together, these pairwise data yield a strong but not significant trend that individuals with access to cleaners have a lower stress response than individuals without access to a cleaner fish (Wilcoxon-Test, n = 14, T = 24, p = 0.07, Fig. 2a).

Independent samples from same reef patch: Males that were caught after the addition of a cleaner fish had a significantly lower stress response than males that were caught prior to the manipulation (Mann-Whitney-U-Test, m = 6, n = 6, U = 0, p < 0.01, Fig. 2b).

## Discussion

The results from this study generally support the idea that fish clients with access to a cleaning organism are less susceptible to stress than clients without access. This is particularly true for chromis, where both cleaner fish and cleaner shrimps had a significant effect. While the result for cleaner shrimps should be confirmed in a future study with larger sample sizes, it emphasises the need for future studies on cleaner shrimps in general. As it stands, there is not even quantitative information on which species visit shrimps how frequently and for how long, though we know that *C. dimidiata *visit cleaner shrimps in our study area (RB, personal observations). A major difference between cleaner fish and cleaner shrimps might be that the fish can exploit clients more easily because they either can take deeper bites of mucus, and/or incur a smaller cost when clients retaliate. More information about the strategic options of cleaner shrimps is needed to evaluate the exact game structure of shrimp-client interactions [33].

The data for anthias are more difficult to interpret. In one analysis we found a significantly lower stress response of individuals with access to cleaners compared to individuals without access, while there was only a trend in the predicted direction in the experiment with matched data from the same individuals. A major problem is the small sample size. We note that the effects of cleaner fish were pretty similar in both chromis and anthias but only significant in chromis. There is no apparent reason why the results should be different for the two species as both are regular clients of cleaner fish at the study site [34].

It is conceivable that individuals who are more easily stressed pay a fitness cost in the long run, since stress has detrimental effects on a number of fitness components such as growth, reproduction, immune function and survival [30,35]. Despite the fact that we have only measured cortisol responses to acute stress, the finding that individuals without access to cleaners are more cortisol-reactive to a stressor than those with access to cleaners, suggests that they are more prone to increase their allostatic load. We did not quantify the ectoparasite loads on our clients. However, there is experimental evidence a) that ectoparasites cause fish to be more susceptible to stress [36], and b) that cleaners reduce the parasite load of clients [17].

**Figure 3:**
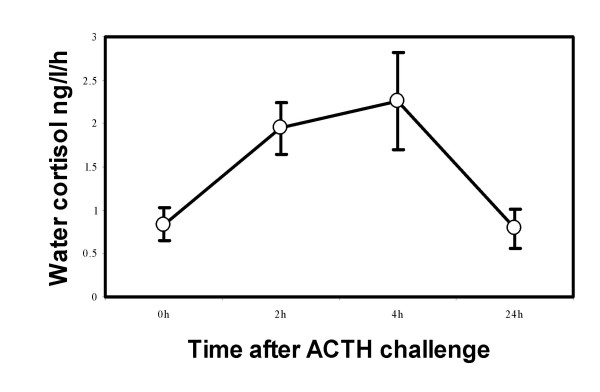
Temporal variation (mean and SEM) of cortisol levels in holding-water of anthias individuals challenged with an intra-peritoneal injection of porcine ACTH. Holding water was changed at each sampling point so that the fish were on the sampled water always for during 1 hour (i.e. there was no accumulation of cortisol in the holding-water with the progress of the experiment).

A weakness of our approach using water samples to assess cortisol levels is that this method does not provide baseline levels. Ideally, we would have liked to take blood samples as well. However, the study was conducted in a National Park where any killing of animals is prohibited, while our study species are too small to survive. Our validation of the water samples using anthias in the laboratory (see methods, Fig. 3) revealed that an ACTH challenge causes cortisol levels that are in a similar range as our field data. The peak in response to the ACTH challenge averages around 2000 pg/l water/h which is somewhat higher than the average response of ca. 1500 pg/l water/h measured for fish without access to cleaners when exposed to stress. If we assume that the ACTH challenge elicited the maximal physiological response than the levels reported in Fig. 1 were ca. 50% for client fish pre-exposed to a cleaner and ca. 75% for fish that had no prior exposure to cleaners before the capture and confinement stress.

A major challenge in studies on mutualism in general and cleaning interactions in our case has been to quantify the costs and benefits associated with interactions [37]. Although, in principle, each of these costs and benefits can be investigated qualitatively, it is difficult to quantify all of them in a single fitness currency to obtain a net result. The measurement of the stress response may provide such a currency, since it gives an indication of the net effect of cleaning on client health, reflecting all costs and benefits mentioned in the introduction. While several cleaner fish removal experiments [6,8,9] show that reef fish can survive without access to cleaning organisms, our endocrine data indicate that they may not be as fit. And this view is supported by recent evidence that if removal experiments are conducted over periods of several month, a decline in species diversity eventually emerges [11,12]. While many studies report apparent benefits of cleaning interactions to clients, the present results are the first ones that suggest that the benefits indeed also exceed the costs of the interactions under natural conditions. The shortcoming of our field study is that we could not control all important parameters like parasite infections and the percentage of time each client spent with cleaners; so the evidence remains correlative. However, our research question is all but impossible to address under laboratory conditions, where cleaners quickly start to exploit clients in the absence of the right balance with respect to parasite re-infection rates and to cleaner-to-client ratios [38].

## Conclusion

In conclusion, we found that cortisol excretion rates in a stress situation, consisting of capture, transport and confinement, differed between reef fish with access to cleaning organisms and reef fish without such access. We propose that the use of confinement stress and cortisol excretion rates, as a measure that provides a correlate of fitness, may turn out to be a sensitive method to determine mutualistic relationships in small reef fish. However, for a more comprehensive interpretation of our results, further studies are necessary and should progress along two lines of research. First, the cortisol excretion rates in response to capture and confinement as a correlate of an individual's fitness should be further evaluated. Second, studies on other interspecific interactions using this methodology will yield a bigger picture on whether apparently mutualistic interactions consistently reduce cortisol levels in stress responses of the actors. If so, this would finally allow a comprehensive analysis of the nature (mutualistic, commensalistic or parasitic) of interspecific interactions in general.

## Methods

### Study site and cleaning organisms

The study was conducted during a six week period from end of May through the beginning of July 1998 at Mersa Bareika, Ras Mohammed National Park, Egypt. In the shallow waters of this area, incoming sand from the (usually dry) riverbeds led to the formation of reef patches that are isolated from each other by sandy areas. The study area comprises a number of small reef patches in shallow water (2–6 m). The reef patches chosen for the study were separated by at least five meters of sand from neighbouring patches. Their estimated respective volumes varied between 3.4–22.3 m^3 ^for anthias and 0.8–14.6 m^3 ^for chromis (see [11] for measurement methods). Both client species do not switch between patches [34]. Neither cleaner fish nor cleaner shrimps are present on all reef patches [[11], RB, unpubl. data]. The cleaner fish *L. dimidiatus *shows strong site fidelity (a fact that prompted the term 'cleaning station') and the same appears to be true for cleaner shrimps: Bshary (unpubl. data) found them repeatedly at the same sites, disappearances and discovery of new sites being rare events. In conclusion, we found natural variation within our client species with respect to access or no access to cleaner organisms. While we did not make observations on cleaning interactions for the present study, every single cleaner fish observed in previous studies at the same site interacted with both client species, typically more often than with any other client species [[34], RB, unpubl. data]. We could therefore safely assume that the presence of a cleaner fish meant that anthias and chromis were being cleaned. *L. dimidiatus *is the main cleaner fish at the site. In addition, there are cleaning shrimps, *Periclimenes longicarpus, Urocaridella sp*. and *Stenopus hipsidus*. Cleaner shrimps have not yet been studied in detail at the study site. We only know from personal observations that they all clean both anthias and chromis. For the present study, we only chose reef patches that had either zero or one cleaner fish present. In the experiment that manipulated the presence of cleaner fish, these solitary cleaner fish were transferred from one study patch to another one (see below). We did not conduct experiments involving cleaner shrimp translocations. We neither counted the number of cleaner shrimps present at the patches where we collected clients to study the impact of cleaner shrimps on client stress response. The most abundant shrimp, *Urocaridella sp*., lives in groups of more than 100 individuals in our study area. In conclusion, our reef patches with cleaner shrimps had many potential cleaning organisms present.

### Stress response of *C. dimidiata*

On eight different days, we caught a total of 20 individual *C. dimidiata *from 7 reef patches without cleaner fish and 20 *C. dimidiata *from 7 reef patches that had a cleaner present. In addition, 6 fish were caught from 2 reef patches with cleaner shrimps present. Each reef patch was visited only once and no more than three fish were caught. So on each day and during the same dive we caught individuals from 2 patches. The individuals caught at the first patch were held in Plexiglas tubes while we caught the individuals from the second patch. We scheduled the catching in a way that we had almost equal numbers per treatment group that were caught first and second (cleaner fish: 4 first, 3 second; cleaner shrimp: 1 first, 1 second; no cleaning organisms: 3 first, 4 second). The fish were brought into a field station laboratory near the field site (50–300 m away from the sites of capture), and each of them was subjected to a confinement stress consisting of 60 minutes in isolation in a small aquarium (10 × 10 × 20 cm), containing 500 ml of seawater. Water was added (50 ml) after 20 and 40 minutes, to renew oxygen. At the end, the fish were released at their site of capture and the water was used for assaying cortisol levels.

### Stress response of *P. squamipinnis*

The design of data collection for the anthias differed from the data collected on chromis, reflecting differences in population structure and sexual dimorphism between the two species. Chromis occurs even on the smallest reef patches while anthias usually occur only on reef patches above a certain size. Chromis is thus particularly suitable for sampling of many different reef patches. Anthias, on the other hand, can be very abundant on a few reef patches and are easier to catch with a barrier net. They are thus more suitable for recapture studies. In addition, anthias are sexually dimorph while chromis are not. Our chromis data therefore represent an unknown mixture of male and female fish, while we decided to focus on the males in anthias. On a given day, depending on local abundance and catching success 4–10 males were caught from one reef patch. Catching duration was restricted to a maximum of 45 min to avoid between-day variation in this parameter, which constitutes one of the stress inducing factors. Males were caught from two reef patches that were without *L. dimidiatus *or cleaner shrimps and from three reef patches with cleaner fish but with no shrimps present. Cleaner shrimps had originally occupied one of the reef patches without cleaning organisms and two of the reef patches with one cleaner fish present but they had been removed 2–4 weeks prior to the experiment. Removal of shrimps required a week of daily visits and catching of 120 to 340 *Periclimenes *and 0 to 7 *Stenopus *and occasional additional removal until the end of the experiments. Thus, while it is unlikely that we removed every single cleaner shrimp, we are confident that we reduced their numbers in an ecologically significant way.

The anthias were marked before being released by dorsal fin clipping, a different one for each individual from the same reef patch. *L. dimidiatus *were removed from the reef patches the day after the males had been caught and introduced to the ones that had been without. This delay in cleaner fish transfer has two reasons. We did not want to disturb the anthias prior to the experiment and we wanted to transfer cleaners in the morning to give them a full day to explore their new environment and find a suitable hide for the night. A fortnight after the transfers, we tried to catch the same anthias males again to repeat the sampling. This experiment worked only partly, since in two reef patches from which the cleaner had been removed, a new cleaner had immigrated before our attempting to catch the anthias again. This was due to the unfortunate coincidence of our experiment being conducted during a settlement phase of young cleaners ending their pelagic stage. We therefore excluded these two patches from further sampling. In addition, only 50% of the fish collected during the second catching visits were recaptures, the other 50% being males caught for the first time (there were more males on the reef patches than were caught on each day). The sample size of individuals that were sampled under both conditions thus became relatively small (n = 14).

We conducted two further analyses with the anthias data. First, in analogy to the chromis data, we checked whether there was a difference between individuals caught from reef patches with and without cleaner organisms. For this analysis, we only used data from catches on our first visit to each reef patch but not the data after manipulations. For this data set, we could include samples of anthias males from two more reef patches that had cleaner shrimps but not *L. dimidiatus *present. A second analysis was made on data collected from a single reef patch. Here, the stress responses of individuals that were only caught the first time (while cleaners were present) were compared with the stress responses of other individuals that were only caught the second time (after cleaners had been removed).

The water samples for *P. squamipinnis *were collected and processed in the same way as the samples of *C. dimidiata*.

### Hormone essays

Assaying steroid hormones from fish holding-water is a non-invasive method recently proposed that has been successfully used in different species [39-42]. The validity of holding-water steroid concentrations as a measure of cortisol secretion levels is based on the following facts: (1) that the release of steroid conjugates in the water is closely associated to specific biologically relevant events (e.g. exposure to a stress stimuli); (2) that the administration of trophic hormones (e.g. gonadotrophin) or of hypothalamic releasing factors that control the production of trophic hormones (e.g. gonadotrophin-releasing hormone) substantially increase the concentrations of specific steroids (e.g. sex steroids) in the water; and (3) that the pattern of steroid release in the water reflects the pattern of secretion into the plasma [38,43,44]. Contrary to circulating concentrations of cortisol taken at a certain point in time, holding-water steroid measurements represent a temporal integration of the cortisol levels that have been in circulation and that have been transferred to the water both by excretion (via urine and the faeces) and by diffusion through the gills [45]. Therefore, holding- water measures are more conservative and less vulnerable to spurious time fluctuations of cortisol levels. In order to validate this method for Anthias and also to demonstrate that we are measuring a stress response to confinement and not baseline levels, we have challenged individual *Pseudanthias squamipinnis *in the lab with adrenocorticotropic hormone (ACTH, Sigma A-6303 ; 0,023 IU/g body weight) and measured the cortisol response curve in the water (Fig. 3). Water was exchanged every hour and each sample analysed for cortisol content. The results show an increase in cortisol immunoreactivity in holding-water in response to the physiological challenge with ACTH, reaching values comparable to those obtained with the confinement stress protocol used (see results).

Water was filtered from each aquarium through an C18 solid phase extraction cartridge (500 mg, Merck), previously activated with 2 × 5 ml ethanol followed by 2 × 5 ml distilled water, and adsorbed material was eluted with 2 × 2 ml ethanol (39) stored at -20°C and shipped to Portugal. Free and conjugated steroids (sulphates and glucuronides) were extracted (see (41) for the extraction protocol) and the fractions for each sample pooled and radioimmunoassayed for total cortisol as an indicator of the stress status of each individual. The cortisol assay cross reactions were: 54% with 11-desoxycortisol; 10% with cortisone; 0.05% with 11-hydroxi-testosterone and <0.001% with testosterone.

### Statistics

Non-parametric statistics were applied because of small sample sizes. We used the statistical package SPSS for UNIX 6.1. All tests were two-tailed.

## Competing interests

The author(s) declare that they have no competing interests.

## Authors' contributions

RB and RFO designed the study and wrote the ms, RB collected the field data in Egypt, TO conducted the validation for anthias in Lisbon, and AVMC the hormonal analyses. All authors read and approved the final version.
